# Artificial intelligence-derived neurofibrillary tangle burden is associated with antemortem cognitive impairment

**DOI:** 10.1186/s40478-022-01457-x

**Published:** 2022-10-31

**Authors:** Gabriel A. Marx, Daniel G. Koenigsberg, Andrew T. McKenzie, Justin Kauffman, Russell W. Hanson, Kristen Whitney, Maxim Signaevsky, Marcel Prastawa, Megan A. Iida, Charles L. White, Jamie M. Walker, Timothy E. Richardson, John Koll, Gerardo Fernandez, Jack Zeineh, Carlos Cordon-Cardo, John F. Crary, Kurt Farrell, The PART working group

**Affiliations:** 1 Department of Pathology, Icahn School of Medicine at Mount Sinai, 1 Gustave L. Levy Place, New York, NY 10029 USA; 2 Department of Artificial Intelligence and Human Health, Nash Family Department of Neuroscience, Ronald M. Loeb Center for Alzheimer’s Disease, Friedman Brain Institute, Neuropathology Brain Bank and Research CoRE, Icahn School of Medicine at Mount Sinai, 1 Gustave L. Levy Place, Box 1194, New York, NY 10029 USA; 3 Department of Psychiatry, Icahn School of Medicine at Mount Sinai, New York, NY USA; 4 New York University McSilver Institute for Poverty Policy and Research, New York, NY USA; 5 Center for Computational and Systems Pathology, Icahn School of Medicine at Mount Sinai, New York, NY USA; 6 Department of Pathology, University of Texas Southwestern Medical Center, Dallas, TX USA

**Keywords:** Tauopathy, Alzheimer’s disease, Primary age-related tauopathy, Neurofibrillary tangle, Digital pathology, Convolutional neural network, Deep learning, Computer vision

## Abstract

Tauopathies are a category of neurodegenerative diseases characterized by the presence of abnormal tau protein-containing neurofibrillary tangles (NFTs). NFTs are universally observed in aging, occurring with or without the concomitant accumulation of amyloid-beta peptide (Aβ) in plaques that typifies Alzheimer disease (AD), the most common tauopathy. Primary age-related tauopathy (PART) is an Aβ-independent process that affects the medial temporal lobe in both cognitively normal and impaired subjects. Determinants of symptomology in subjects with PART are poorly understood and require clinicopathologic correlation; however, classical approaches to staging tau pathology have limited quantitative reproducibility. As such, there is a critical need for unbiased methods to quantitatively analyze tau pathology on the histological level. Artificial intelligence (AI)-based convolutional neural networks (CNNs) generate highly accurate and precise computer vision assessments of digitized pathology slides, yielding novel histology metrics at scale. Here, we performed a retrospective autopsy study of a large cohort (*n* = 706) of human post-mortem brain tissues from normal and cognitively impaired elderly individuals with mild or no Aβ plaques (average age of death of 83.1 yr, range 55–110). We utilized a CNN trained to segment NFTs on hippocampus sections immunohistochemically stained with antisera recognizing abnormal hyperphosphorylated tau (p-tau), which yielded metrics of regional NFT counts, NFT positive pixel density, as well as a novel graph-theory based metric measuring the spatial distribution of NFTs. We found that several AI-derived NFT metrics significantly predicted the presence of cognitive impairment in both the hippocampus proper and entorhinal cortex (*p* < 0.0001). When controlling for age, AI-derived NFT counts still significantly predicted the presence of cognitive impairment (*p* = 0.04 in the entorhinal cortex; *p* = 0.04 overall). In contrast, Braak stage did not predict cognitive impairment in either age-adjusted or unadjusted models. These findings support the hypothesis that NFT burden correlates with cognitive impairment in PART. Furthermore, our analysis strongly suggests that AI-derived metrics of tau pathology provide a powerful tool that can deepen our understanding of the role of neurofibrillary degeneration in cognitive impairment.

## Introduction

Neurofibrillary tangles (NFT), inclusions composed of toxic hyperphosphorylated forms of the microtubule-associated protein tau (p-tau), are the defining neuropathological feature of a category of neurodegenerative diseases termed tauopathies [1, 2]. This large group of diseases includes primary age-related tauopathy (PART) [3], Alzheimer’s disease (AD) [1], argyrophilic grain disease (AGD) [4], frontotemporal lobar degeneration (FTLD) [5], and chronic traumatic encephalopathy (CTE) [6]. PART describes a neuropathologic continuum observed in the brains of elderly individuals containing p-tau pathology in the absence of or with mild amounts of amyloid-beta peptide (Aβ). Subjects with a Consortium to Establish a Registry for Alzheimer's Disease (CERAD) neuritic plaque severity score of zero are considered PART definite while those with a score of one are considered PART probable. Clinically, those with PART may or may not have cognitive impairment [3, 7], raising the possibility that other factors (e.g. cerebrovascular disease) play a role. For these reasons, studying PART provides an opportunity to assess age-related neurodegenerative processes that contribute to cognitive impairment. The relationship between cognitive impairment in PART and NFT burden is currently not well understood [7]. For example, non-impaired individuals can have a significant NFT burden, complicating our understanding of the contribution of such brain changes to symptomatology [3, 7]. Conversely, it is well understood that NFTs accumulate with age and that individuals who are older are more likely to have cognitive decline [8]. Thus, the age-independent relationship between NFT burden and cognitive impairment in PART remains unclear. One approach to improving our understanding of the complex relationship between NFT burden, aging, and clinical presentation is by leveraging more precise quantification of histologic features.

Prior to the introduction of computational-based approaches to neuropathology, the Braak tau staging system was the most prevalent method of measuring pathological p-tau burden in research and remains so in the clinical setting [9]. While this method has its strengths, it is inherently semi-quantitative, modestly reproducible, and subject to rater bias, leading to inconsistencies between evaluators and institutions [10–14]. Further, the Braak staging system was developed for assessment of p-tau pathology in the context of AD and has not been sufficiently validated in specifically Aβ-negative subjects. The Braak staging system is based on hierarchical neuroanatomical spread and not the degree of p-tau burden in specific brain regions [9, 12]. Despite it being a reflection of p-tau topographic distribution, it is often used as a proxy for assessing the magnitude of neurofibrillary degeneration due to lack of convenient alternatives [15–18]. Consequently, in PART, which minimally advances outside of the medial temporal lobe, two cases with large differences in NFT burden have the same Braak stage. We have found that Braak staging has suboptimal clinicopathologic predictive power in Aβ-negative individuals [19]. Thus, there is a need for better quantitative approaches to assessing p-tau burden [20–23].

Recent developments in whole slide digitization allow the use of computational approaches to precisely assess and quantify neuropathological features. This includes measuring histological staining intensity (e.g., positive pixels), which we have previously deployed in the context of hippocampal tissue sections immunohistochemically-stained for p-tau [19]. However, this approach fails to distinguish between critical structural and morphological features that could assist in our understanding of the relationship between neuropathology and antemortem clinical symptomatology. Furthermore, this method relies on human defined pixel color ranges and intensities, and is thus vulnerable to biases of variable effects of formalin fixation on tinctorial properties [24]. An alternative approach is to utilize deep-learning based models such as convolutional neural networks (CNNs). CNNs can be trained to generate meaningful histologic metrics on whole slide images (WSIs) to assist in feature quantification [25], classification [26], or segmentation [27]. There is a growing literature of successful applications of CNNs and other deep learning methods in neuropathology [28–33]. Previous CNN based approaches to neuropathology immunohistochemistry (IHC) have proven successful at classifying tauopathies based on p-tau lesions [32], detecting and categorizing Aβ lesions [28, 34], and calculating alpha-synuclein burden from submandibular gland biopsy [33].

Signaevsky et al*.* 2019 trained a SegNet [35] semantic segmentation model on WSIs of hippocampal tissue immunohistochemically stained for p-tau and annotated by expert neuropathologists [29]. The training dataset was a set of manual segmentations of NFT’s, excluding partial neurites lacking connection to the soma or hillock. The model achieved an F1 score of 0.85 for NFT segmentation in PART cases [29]. Unlike state of the art computational approaches to assessing p-tau burden, Segnet is able to discriminate between the pixels in a WSI that specifically represent NFTs from pixels representing glial-tau inclusions, neuropil threads, background tissue, and artifacts [29]. Using this model, it is possible to obtain quantitative metrics, such as NFT number and size, as well as spatial information about each NFT in the image. Here, we leverage this model to extract AI-derived metrics of NFT hippocampal neuropathology from a cohort of 706 autopsy-confirmed donors with PART. We then compared how our AI-derived metrics of NFT burden compared with positive-pixel counts and Braak staging in predicting cognitive impairment with and without correcting for age. We also introduce a novel histologic phenotype of NFT-clustering, which is a graph-theory based measure of NFT spatial distribution in the medial temporal lobe.

## Methods

### Patient samples

Scanned digital images of formalin-fixed paraffin embedded (FFPE) tissue sections from the hippocampus as well as fresh-frozen tissue from the frontal cortex were derived from autopsy brains from a subset of individuals from a previously described collection [16]. Clinical inclusion criteria included being cognitively normal or having a diagnosis of mild cognitive impairment (MCI) or dementia with a recorded clinical dementia rating (CDR), Mini-Mental State Examination (MMSE), or postmortem clinical chart review CDR score [36]. CDR and MMSE scores were used to assign subjects into either cognitively normal or cognitively impaired groups. Individuals who had a CDR score of 0.5 or above or MMSE score below 26 were considered to be cognitively impaired, while subjects with a CDR score of 0 or MMSE score 26 or above were considered cognitively normal. If an individual had both MMSE score and CDR score, the most recent score was used, and if both scores were given on the same date, the CDR score was used.

Comprehensive neuropathological assessments were performed at the contributing institutions. Neuropathological exclusion criteria consisted of other neurodegenerative diseases including AD, Lewy body disease, progressive supranuclear palsy (PSP), corticobasal degeneration (CBD), chronic traumatic encephalopathy (CTE), Pick disease, Guam amyotrophic lateral-sclerosis-parkinsonism-dementia, subacute sclerosing panencephalitis, globular glial tauopathy, and hippocampal sclerosis. Data pertaining to Braak stage, CERAD, Lewy body pathology (incidental), cerebrovascular disease, infarcts (vascular brain injury), microinfarcts, and argyrophilic grains, were derived from neuropathologic studies performed at respective centers. Incidental Lewy body pathology was defined as the presence of rare to sparse Lewy bodies (as assessed at the providing center) in the absence of movement disorder. The presence of aging-related tau astrogliopathy (ARTAG) was determined on p-tau immunohistochemical stains described below.

### Immunohistochemistry

Immunohistochemistry and hematoxylin & eosin (H&E) stains were performed on 5 μm FFPE sections mounted on positively charged slides and dried overnight at room temperature. IHC was performed on a Leica Bond III automated stainer, according to the manufacturer’s protocols (Leica Microsystems, Buffalo Grove, IL, USA) using antibodies to hyperphosphorylated tau (p-tau, AT8, 1:1000, Fisher Scientific, Waltham, MA, USA) and Aβ (Aβ, 6E10, 1:1000, Covance, Princeton, NJ, USA). For each set of slides, a known severe AD case was included as a batch control and compared to ensure uniform staining across all samples.

### Genetic analysis

High-throughput isolation of DNA was performed using the MagMAX DNA Multi-Sample Ultra 2.0 Kit on a KingFisher Flex robotic DNA isolation system (Thermofisher, Waltham, MA) according to manufacturer protocol. Briefly, 20–40 mg of fresh frozen brain tissue was placed into a deep-well plate and treated with 480 ul of Proteinase K mix (Proteinase K, Phosphate Buffered Saline [pH 7.4], Binding Enhancer) and incubated overnight at 65 °C at 800 rpm on a shaking plate. Genomic DNA was isolated and purified using magnetic particles. DNA quality control was performed using a nanodrop spectrophotometer (concentration > 50 ng/ul, 260/280 ratio 1.7–2.2). Genotyping was performed using single nucleotide polymorphism (SNP) microarrays (Infinium Global Screening Array v2.4. or the Infinium OmniExpress-24, Illumina, San Diego CA). Raw genotype files were converted to PLINK-compatible files using GenomeStudio software (Illumina, San Diego CA). MAPT haplotype was determined using the rs8070723 H2 tagging SNP and APOE genotype was determined using the rs429358 rs7412 tagging SNPs. For analyses, the APOE status was collapsed into a binary variable of the presence or absence of APOE ε4.

### NFT burden calculation and slide level annotation

**Fig. 1:**
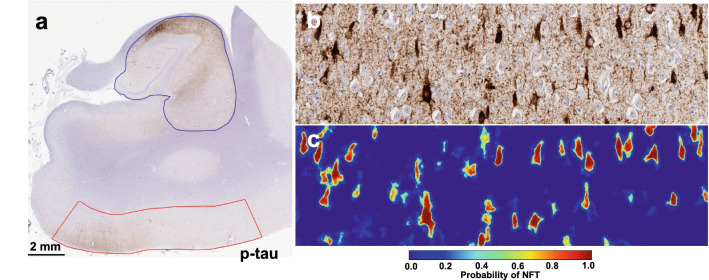
Detection of neurofibrillary tangles (NFT) in phospho-tau (AT8) immunohistochemically stained whole slide images (WSI). **a** Example of a hippocampal WSI immunohistochemically stained for phosphorylated-tau (AT8). The hippocampus proper (blue) and entorhinal region (red) were manually segmented. **b** High-power (20x) representative image of the hippocampal CA2 subfield showing p-tau positive neurofibrillary tangles. **c** Corresponding output of above image passed through semantic segmentation model that identifies NFT. Each pixel value corresponds to the probability that it represents an NFT

Neurofibrillary tangles (NFT) were semantically segmented from whole slide images (WSI) (Fig. 1a–c) using a SegNet model architecture, detailed in Signaevsky et al*.* 2019, which was trained on annotations performed by expert neuropathologists on 2221 NFTs from 14 different WSIs. For each slide, the model calculated NFT number, size, and location. WSIs were neuroanatomically segmented into the hippocampus proper (i.e., dentate gyrus, cornu ammonis, and subiculum) and the adjacent entorhinal cortex region, which variably includes posterior portions of the parahippocampal gyrus and the (trans-)entorhinal region or lingual gyrus (Fig. 1a) using Aperio ImageScope software. NFT counts were calculated for each region as the number of NFTs divided by the area of the region. AI-derived NFT positive pixel density was calculated as the sum of the area of all NFTs in a region divided by the area of the region. For standard positive pixel calculations, staining was measured in the hippocampus proper and entorhinal cortex separately and together using a modified version of the Aperio positive pixel count (Version 9) based on the intensities of the positive control sample in each batch to determine the area of immunoreactivity. Positive pixel counts were normalized using the number of positive pixel counts to the total area creating a 0–1 p-tau burden scale.

### Mean clustering coefficient calculation

To estimate the degree of NFT clustering for a given WSI, we represented the spatial distribution of NFTs as a network and calculated the mean clustering coefficient. The center coordinate of each NFT is represented as a two-dimensional point cloud fed into a kd-tree and queried all points within a given radius, *r*. Thus, the spatial distribution of NFT for a given WSI is represented as a graph where each NFT is a node and its neighbors are the other NFTs within a distance of *r* (Fig. 5a). There is no standard metric of inter-NFT distance, therefore we created graphs over multiple values of *r* from 100 (50.66 microns) to 5000 pixels (2533 microns) in 100 pixel intervals. To correct for the whole slide NFT burden in this calculation, all statistics for this metric included the total number of NFTs as a nuisance variable.

### Statistical analysis

All statistics were carried out via the statsmodels library in Python [37]. Data was visualized using the ggplot2 package in project R [38]. Descriptive statistics were used to identify differences between the cognitively normal and cognitively impaired PART groups for clinical, pathological, and genetic variables. Differences were detected using chi-square. A t-test was performed to determine if age differed significantly between normal and cognitively impaired groups. A multivariable model was created to determine to what extent measures of NFT burden (Braak NFT stage, positive pixel count, and AI-based) predict cognitive impairment in PART. Analyses evaluating associations between NFT burden and individual sub-measures of cognitive impairment utilized *t*-test for clinical diagnosis, Spearman rank-order for CDR, and Pearson correlation for MMSE. Age-adjusted models included age as a parameter. All statistical analyses using measures of NFT burden were corrected for multiple comparisons via false discovery rate.

## Results

### Dataset demographics, neuropathologic findings, and genetics

A total of 706 subjects were included in this study (Table 1). The overall mean age was 85.15 with a range of 55 to 110 years. Of these, 362 subjects (mean age 82.96, 168 male, 194 female) had no cognitive impairment (NCI) and 344 subjects (mean age 87.45, 161 male, 183 female) had some degree of cognitive impairment (CI). The CI group was significantly older than the NCI group (*p* < 0.0001). In our genetic analysis, we found no significant interaction between cognitive impairment and presence of ε2 *APOE* allele, ε4 *APOE* allele, or *MAPT* haplotype distribution.

**Table 1:** Summary of cohort data

		Overall	Cognitive status		*p*
			Impaired	Normal	
*Demographics*					
	Total (M/F)	706 (329/377)	344 (161/183)	362 (168/194)	0.9766
	Average age at death (standard deviation)	85.15 (10.27)	87.45 (8.91)	82.96 (10.98)	< 0.0001*
*Neuropathologic data*					
	Presence of hippocampal ARTAG (%)	166 (26.90%)	96 (31.27%)	70 (22.58%)	**0.0191**
	*Braak NFT Stage*				0.4282
	0	64 (9.07%)	33 (9.59%)	31 (8.56%)	
	I	111 (15.72%)	46 (13.37%)	65 (17.96%)	
	II	189 (26.77%)	89 (25.87%)	100 (27.62%)	
	III	186 (26.35%)	93 (27.03%)	93 (25.69%)	
	IV	126 (17.85%)	69 (20.06%)	57 (15.75%)	
	V	29 (4.11%)	13 (3.78%)	16 (4.42%)	
	*CERAD Score*				0.4829
	C0	565 (80.03%)	265 (77.03%)	300 (82.87%)	
	C1	111 (15.72%)	59 (17.15%)	52 (14.36%)	
*Genetics Data*					
	Presence of E2 APOE allele	22 (22.45%)	12 (30.77%)	10 (16.95%)	0.1746
	Presence of E4 APOE allele	15 (15.31%)	3 (7.69%)	12 (20.34%)	0.1569
	*APOE Genotype*				0.3225
	APOE2,2	3 (3.06%)	2 (5.13%)	1 (1.69%)	
	APOE2,3	17 (17.35%)	9 (23.08%)	8 (13.56%)	
	APOE2,4	2 (2.04%)	1 (2.56%)	1 (1.69%)	
	APOE3,3	63 (64.29%)	25 (64.10%)	38 (64.41%)	
	APOE3,4	10 (10.20%)	2 (5.13%)	8 (13.56%)	
	APOE4,4	3 (3.06%)	0 (0%)	3 (5.08%)	

Group comparisons are conducted via chi-squared test *except for average age at death which is a *t*-test. Bold *p*-values indicate *p* < 0.05

Neuropathologic case review found 166 subjects (26.9%) exhibited hippocampal age-related tau astrogliopathy (ARTAG). Comparing between the groups, we found CI had significantly higher rates of ARTAG than NCI (31.27% vs 22.58%, *p* = 0.019). Considering that both ARTAG and CI are more prevalent in the elderly, we found after age adjustment via Cochran-Mantel–Haenszel method with two-level stratification there was no longer a significant association between ARTAG and CI (pooled OR: 1.42, *p* = 0.058). There was no significant statistical difference in Braak NFT stage scores between the two groups (NCI: mean 2.35, stdev 1.30; CI: mean 2.46, stdev 1.31; two tailed *t*-test, *p* = 0.27; chi-square test, *p* = 0.43). There were no significant differences in the distribution of CERAD score between the groups (NCI: mean 0.15, stdev 0.37; CI: mean 0.19, stdev 0.40; chi-square test, *p* = 0.48).

### Tau burden

**Fig. 2:**
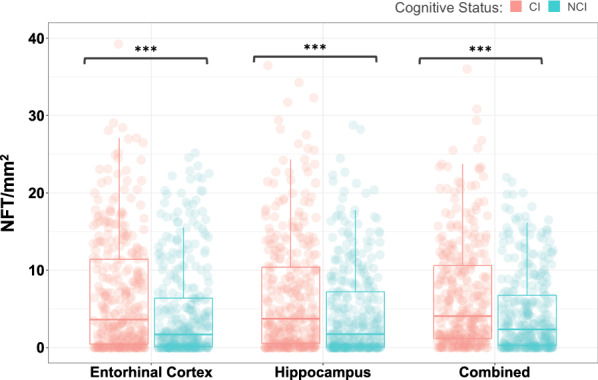
Increased neurofibrillary tangle (NFT) counts in cognitively impaired subjects. NFT densities are shown split by cognitive status, cognitively impaired (CI) and no cognitive impairment (NCI). NFT counts of the entorhinal cortex, hippocampus, and both regions combined are presented. Triple asterisks (***) denote *p* < 0.0001 based on a two-sample *t*-test between groups. Two-way analysis of variance yielded a F-statistic of 58.99

In our main unadjusted analysis of tau burden as a predictor of cognitive status (Table 2), we found that the Braak NFT stage was not a significant predictor of cognitive impairment (OR 1.09, *p* = 0.2769). However, both AI-detected NFT counts and AI-detected NFT positive pixel density were significant predictors of cognitive impairment in the entorhinal cortex (counts, OR 1.38, *p* = 0.0001; pixels, OR 1.32, *p* < 0.0001), hippocampus (counts, OR 1.40, *p* = 0.0001; pixels, OR 1.35, *p* < 0.0001), and combined regions (counts, OR 1.45, *p* < 0.0001; pixels, OR 1.40, *p* < 0.0001) (Fig. 2). Standard p-tau immunoreactivity positive pixel count was also a significant predictor of cognitive impairment in the entorhinal cortex (OR 1.29, *p* = 0.0039), hippocampus (OR 1.42, *p* = 0.0002), and combined regions (OR 1.39, *p* = 0.0002).

**Table 2:** Odds of being cognitively impaired at death based on p-tau metric

Measure of p-tau burden	Unadjusted			Age adjusted		
	OR	95% CI	*p*	OR	95% CI	*p*
*Classical staging*						
Braak NFT stage	1.09	0.94–1.26	0.2769	0.89	0.75–1.05	0.1603
*Positive pixel count*						
Entorhinal Cortex	1.29	1.09–1.52	**0.0039**	1.15	0.96–1.37	0.1467
Hippocampus	1.42	1.20–1.69	**0.0002**	1.01	1.01–1.46	0.0666
Combined	1.39	1.17–1.65	**0.0002**	1.21	1.01–1.45	0.0678
*AI-detected NFT counts*						
Entorhinal Cortex	1.38	1.18–1.61	**0.0001**	1.25	1.06–1.47	**0.0373**
Hippocampus	1.40	1.20–1.64	**0.0001**	1.22	1.04–1.44	0.0595
Combined	1.45	1.24–1.70	** < 0.0001**	1.28	1.08–1.51	**0.0373**
*AI-derived NFT positive pixel density*						
Entorhinal Cortex	1.32	1.13–1.54	** < 0.0001**	1.19	1.01–1.39	0.0666
Hippocampus	1.35	1.15–1.58	** < 0.0001**	1.17	0.99–1.38	0.0847
Combined	1.40	1.19–1.63	** < 0.0001**	1.20	1.03–1.43	0.0598
*Network based metric*						
Average clustering coefficient	1.27	1.08–1.49	**0.0039**	1.16	0.98–1.35	0.1162

Statistics were corrected for multiple comparisons using false discovery rate. Bold *p*-values indicate *p* < 0.05

**Fig. 3:**
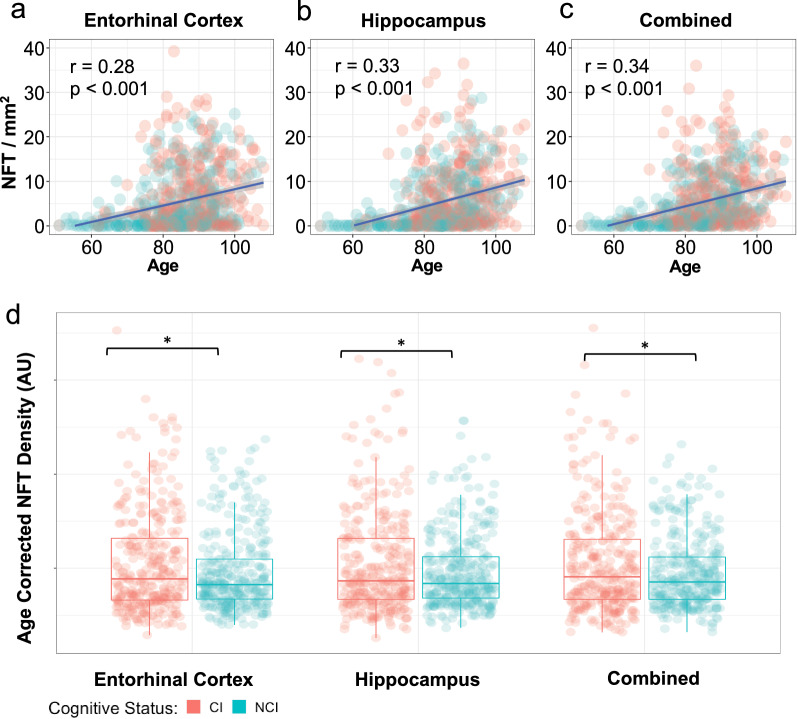
AI-detected NFT counts by region with respect to age and cognitive status. **a**–**c** Relationship between NFT counts and age, grouped by cognitive status in Entorhinal Cortex (**a**), Hippocampus (**b**), and combined (**c**). Pearson correlation values between age and region’s NFT density are shown with associated p value. **d** Age adjusted NFT density group difference by region. Asterisks denote *p* < 0.05 based on a two-sample *t*-test between groups. Two-way analysis of variance yielded a F-statistic of 4.23

Similarly, in our age-adjusted analysis of tau burden as a predictor of cognitive status (Table 2), we found that the Braak NFT stage was not a significant predictor of cognitive impairment (OR 0.89, *p* = 0.1603). Age-corrected AI-detected NFT counts were a significant predictor of cognitive impairment in the entorhinal cortex (OR 1.15, *p* = 0.0373) and combined regions (OR 1.28, *p* = 0.0373), but not the hippocampus (OR 1.22, *p* = 0.0595) (Fig. 3D). In contrast, age-corrected AI-detected NFT positive pixel density and age-corrected standard positive pixel count were not a significant predictor of cognitive impairment in the entorhinal cortex (AI-pixel, OR 1.19, *p* = 0.0666; standard pixel, OR 1.15, *p* = 0.1467), hippocampus (AI-pixel, OR 1.17, *p* = 0.0847; standard pixel,OR 1.01, *p* = 0.0666), or combined regions (AI-pixel, OR 1.20, *p* = 0.0598; standard pixel, OR 1.21, *p* = 0.0678). When comparing AI-detected NFT counts with age (Fig. 3 a-c), we found a significant correlation between NFT counts and age in the entorhinal cortex (r = 0.28, *p* < 0.0001), hippocampus (r = 0.33, *p* < 0.0001), and combined regions (r = 0.34, *p* < 0.0001).

**Fig. 4:**
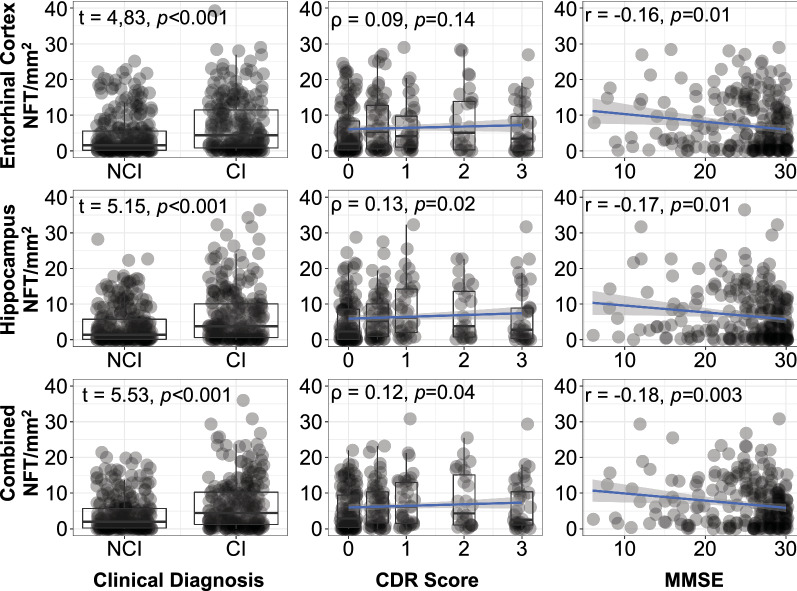
Relationship between NFT counts by region and each individual cognitive variable. In this analysis we used a loose label of cognitive impairment as a composite metric based on MMSE, CDR, or documented clinical history of cognitive impairment. This figure shows the relationships between AI-detected NFT counts by region and each individual clinical variable. (Left column) Two-sample t-tests were performed for documented clinical history of cognitive impairment. (Middle column) Spearman rho correlation was performed between NFT count and CDR score. (Right column) Pearson r correlation was performed between NFT count and MMSE

Detailed breakdown of associations between regional AI-detected NFT counts and each individual clinical variable can be found in Fig. 4. There was a significantly increased (p < 0.001) NFT in cases with a positive clinical diagnosis of cognitive impairment vs those without in all regions and combined. There was a modest yet statistically significant positive correlation between NFT counts and CDR score in the hippocampus (⍴ = 0.13, p = 0.02) and combined regions (⍴ = 0.12, *p* = 0.04) but insignificant in the entorhinal cortex (⍴ = 0.09, *p* = 0.14). There was a significant negative correlation between NFT counts and MMSE score in the entorhinal cortex (r = − 0.16, *p* = 0.01), hippocampus (r = − 0.17, *p* = 0.01), and combined regions (r = − 0.18, *p* = 0.003).

### NFT Spatial Clustering Analysis

**Fig. 5:**
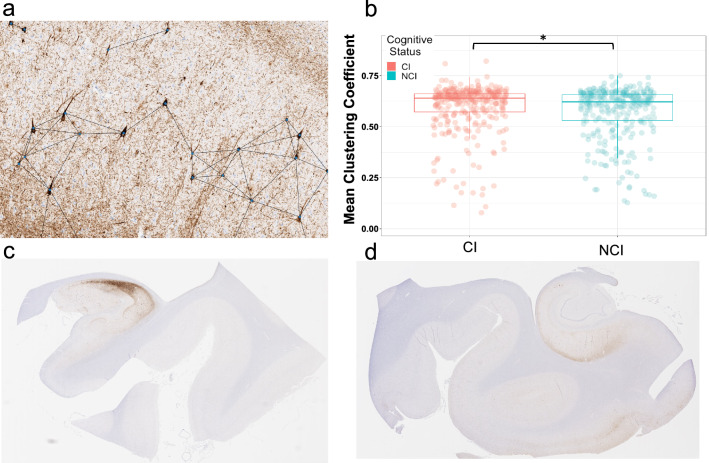
NFT position as a geometric network and subsequent graph metrics. **a** A representation of NFT position as a geometric network. Each NFT is represented as a node in a unidirectional binary graph, where an edge exists between two nodes if the Euclidean distance between them is less than some value *r*. In this figure r = 250 μm. **b** Group comparison of non-cognitively impaired (NCI) vs cognitively impaired (CI) mean clustering coefficient. Asterisk denotes *p* < 0.05. Two-sample *t*-test between groups yielded a t-statistic of  − 2.97 and *p* = 0.0031. **c:** Example of hippocampal whole slide image with high mean clustering coefficient (0.75). **d** Example of hippocampal whole slide image with low mean clustering coefficient (0.47)

**Fig. 6:**
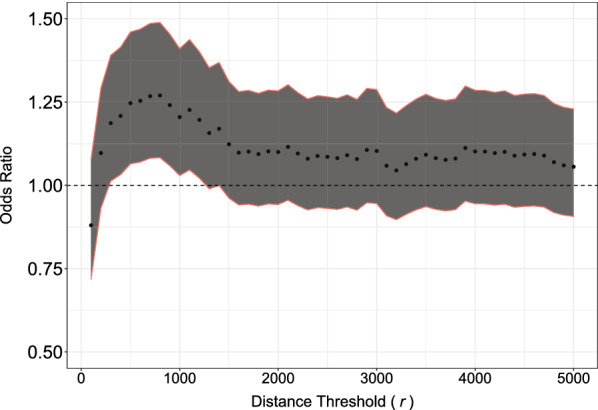
Odds ratio of cognitive impairment on mean NFT clustering coefficient for a range of given distance thresholds. Since the cutoff of *r* in our mean NFT clustering coefficient metric has no ground truth, we tested it across a large range of values. Red lines bounding shaded areas demark the upper and lower bounds of the 95% confidence interval. Mean NFT clustering coefficient significantly predicts cognitive impairment for distance thresholds between 300 px (151.98 microns) and 1200 px (607.92 microns)

In our analysis of NFT clustering, we found that degree of NFT clustering significantly predicted cognitive impairment over a range of distance threshold values (*r*) (Fig. 5 b), with a maximum odds ratio (OR 1.27, *p* = 0.0039) at *r* = 800 px (405.28 microns) (Table 2). We found NFT clustering significantly predicted cognitive impairment across the range of distance threshold values, *r*, between 300 and 1200 pixels (151.98 microns—607.92 microns) (Fig. 6). With age adjustment, mean clustering coefficient did not significantly predict cognitive impairment (OR 1.16, *p* = 0.1162) (Table 2).

## Discussion

Machine learning has emerged as a rigorous and reproducible quantitative approach for assessing neurodegenerative lesions in human autopsy brain tissues, including neurofibrillary tangles and Aβ plaques, key components of AD, aging, and related diseases. It is unclear, however, whether these AI-derived traits are clinically relevant. Improving our ability to assess clinical correlates of neuropathological features, which remain modest even with widely deployed approaches [39], is an important priority. Here we show, in an autopsy cohort of 706 subjects meeting the neuropathological criteria for PART, that AI-derived measurement of NFT burden, derived from digitized WSIs of the hippocampus immunohistochemically stained for p-tau in the medial temporal lobe, significantly predicts antemortem cognitive impairment. This AI classifier greatly outperformed Braak staging, the gold standard approach of NFT burden measurement, which did not predict cognitive impairment in this selected cohort. This supports our previous findings that widely deployed approaches may not fully capture clinically relevant disease burden in brains with PART [19].

While previous digital pathology studies have found correlations between p-tau burden and cognitive impairment [8, 18, 19, 40, 41], this is the first study, to our knowledge, to perform clinicopathologic correlations using AI-assisted NFT counts in a population of non-AD or related disease patients. Previous work using positive pixel counts in p-tau immunohistochemically stained digitized sections have provided a reliable estimate of p-tau burden [19, 42–45], however NFT segmentation via convolutional neural networks (CNNs) gives highly sensitive and specific measurements of NFT burden which are unbiased by neuropil threads or other tau-based pathologic structures [29]. In addition, AI-based CNNs generate novel metrics describing the size, morphology and spatial distribution of NFTs. Notably, of the computational measures of p-tau burden, we found that AI-derived metrics of NFT counts were the only measures to detect an age-independent relationship between NFT burden and cognitive impairment. Thus, we conclude that AI-derived measures of NFT burden are a valuable and precise histologic tool that can be implemented at scale to assess subtle relationships which may underlie clinically relevant signals without requiring the labor of manually counting NFTs on hundreds of WSIs. In summary, studies like this which leverage AI-derived histomics assist in demonstrating the feasibility of deploying such metrics in clinicopathologic correlation studies in neuropathology.

In addition to rapidly quantifying tangle burden on a large dataset of donors, we also introduced a novel metric of NFT mean clustering coefficient which was able to quantify the spatial density of NFTs in a given sample. We found that NFT mean clustering coefficient reliably predicted cognitive impairment in our population of PART patients. This metric provides a novel insight into the distribution of p-tau in a given section, a measure which so far has only been indirectly approximated [46]. We hypothesize the utility of this metric can assist in predicting cognitive impairment in tauopathies which are more focally distributed such as CTE [44, 46]. This approach to measuring disease burden has the theoretical potential to capture mechanisms of p-tau spread through a given region, which is currently under investigation by several other groups [47–54]. Previous work has shown the extent to which graph-based spatial measures can estimate disease burden in histopathology [55]. Of note, Signaevsky et al*.* 2022 found that graph-based metrics of spatial distribution of αα-synuclein lesions had the highest predictive value in diagnosing Parkinson’s disease over all other measures of α-synuclein burden [33]. Future studies will seek to leverage several more AI-generated features of neurodegeneration, including but not limited to tangle shape and morphology, white matter involvement, and other pathological classifiers.

While our study demonstrated a strong correlation between NFT burden and cognitive impairment, there are notable limitations. We designated cognitive status using a weak threshold based on limited available clinical information, including three different measures of cognitive impairment [30]. Correlative studies within prospective cohorts with antemortem neuropsychological assessments would allow for the potential to analyze differential relationships between anatomic subregional vulnerability and specific cognitive domain deficits. Telyan et al*.* 2020 found longitudinal decline within specific cognitive domains in a population of PART patients [56], however it remains unknown what histopathologic features underlie deficits in each domain. Correlative studies within prospective cohorts with antemortem neuropsychological assessments would allow for the potential to analyze differential relationships between anatomic subregional vulnerability and specific cognitive domain deficits. Further, the timeframe under which patients' clinical data were obtained before death was variable, and some may have progressed in this time window. Additionally, the cohort was not population based. For all these reasons, our clinical classification is inherently noisy. While this approach has modest sensitivity for cognitive impairment, we nevertheless found that our measures of NFT burden significantly correlated with each individual cognitive measure independently, demonstrating the utility of this AI-derived metric to detect a signal despite a high degree of noise. Another limitation is the use of coarse neuroanatomical annotations which did not follow subregion boundaries with known selective vulnerability profiles in PART [57, 58]. Follow up studies are ongoing to establish protocols for detailed hippocampal subregion annotations for future analysis, as well as leverage subregion specific p-tau burden metrics in clinicopathologic, genomic, and transcriptomic correlative studies. Further, this study did not account for the contributions of certain pathologic features (e.g., TDP-43, cerebrovascular disease, degree of neuronal loss) relevant to both cognitive impairment and the degree of neurofibrillary degeneration [19, 30, 59, 60]. Thus, future studies are necessary to measure the extent to which our observed associations would remain after accounting for their confounding influence. While this study establishes clinicopathologic correlations between AI-derived measures of NFT burden in a population of PART patients, further studies are required to validate these findings in other populations and tauopathies such as AD, FTLD, and CTE.

In conclusion, here we demonstrate that our AI-derived measures of neurofibrillary degeneration offer a rapid, robust, and reproducible approach to identifying histopathological features which predict antemortem cognitive impairment independently of age. These results support our prior work showing a strong correlation between cognitive impairment and the degree of NFT pathology using positive-pixel counts in the medial temporal lobe in PART. Further, this study demonstrates that AI-derived metrics have the potential to provide novel histologic signatures for clinicopathologic correlation in future studies.

## References

[1] Arriagada PV, Growdon JH, Hedley-Whyte ET, Hyman BT (1992). Neurofibrillary tangles but not senile plaques parallel duration and severity of Alzheimer’s disease. Neurology..

[2] Hernández F, Avila J (2007). Tauopathies. Cell Mol Life Sci.

[3] Crary JF, Trojanowski JQ, Schneider JA, Abisambra JF, Abner EL, Alafuzoff I (2014). Primary age-related tauopathy (PART): a common pathology associated with human aging. Acta Neuropathol (Berl).

[4] Rodriguez RD, Grinberg LT (2015). Argyrophilic grain disease: an underestimated tauopathy. Dement Neuropsychol.

[5] Mohandas E, Rajmohan V (2009). Frontotemporal dementia: an updated overview. Indian J Psychiat.

[6] McKee AC, Stein TD, Kiernan PT, Alvarez VE (2015). The neuropathology of chronic traumatic encephalopathy. Brain Pathol.

[7] Besser LM, Mock C, Teylan MA, Hassenstab J, Kukull WA, Crary JF (2019). Differences in cognitive impairment in primary age-related tauopathy versus alzheimer disease. J Neuropathol Exp Neurol.

[8] Jefferson-George KS, Wolk DA, Lee EB, McMillan CT (2017). Cognitive decline associated with pathological burden in primary age-related tauopathy. Alzheimers Dement.

[9] Braak H, Braak E (1995). Staging of alzheimer’s disease-related neurofibrillary changes. Neurobiol Aging.

[10] Alafuzoff I, Arzberger T, Al-Sarraj S, Bodi I, Bogdanovic N, Braak H (2008). Staging of neurofibrillary pathology in alzheimer’s disease: a study of the brainnet europe consortium. Brain Pathol.

[11] Ball MJ, Murdoch GH (1997). Neuropathological criteria for the diagnosis of alzheimer’s disease: are we really ready yet?. Neurobiol Aging.

[12] Del Tredici K, Braak H (2020). To stage, or not to stage. Curr Opin Neurobiol.

[13] Gertz H-J, Xuereb J, Huppert F, Brayne C, McGee MA, Paykel E (1998). Examination of the validity of the hierarchical model of neuropathological staging in normal aging and Alzheimer’s disease. Acta Neuropathol (Berl).

[14] Brunnström H, Englund E (2011). Comparison of four neuropathological scales for alzheimer’s disease. Clin Neuropathol.

[15] Hamasaki H, Honda H, Okamoto T, Koyama S, Suzuki SO, Ohara T (2016). Recent increases in hippocampal tau pathology in the aging japanese population: the hisayama study. J Alzheimers Dis.

[16] Farrell K, Kim S, Han N, Iida MA, Gonzalez EM, Otero-Garcia M (2022). Genome-wide association study and functional validation implicates JADE1 in tauopathy. Acta Neuropathol (Berl).

[17] Thom M, Liu JYW, Thompson P, Phadke R, Narkiewicz M, Martinian L (2011). Neurofibrillary tangle pathology and Braak staging in chronic epilepsy in relation to traumatic brain injury and hippocampal sclerosis: a post-mortem study. Brain J Neurol.

[18] Gold G, Bouras C, Kövari E, Canuto A, González Glaría B, Malky A (2000). Clinical validity of braak neuropathological staging in the oldest-old. Acta Neuropathol (Berl).

[19] Iida MA, Farrell K, Walker JM, Richardson TE, Marx GA, Bryce CH (2021). Predictors of cognitive impairment in primary age-related tauopathy: an autopsy study. Acta Neuropathol Commun.

[20] Takayama M, Kashiwagi M, Matsusue A, Waters B, Hara K, Ikematsu N (2016). Quantification of immunohistochemical findings of neurofibrillary tangles and senile plaques for a diagnosis of dementia in forensic autopsy cases. Leg Med.

[21] Moloney CM, Lowe VJ, Murray ME (2021). Visualization of neurofibrillary tangle maturity in Alzheimer’s disease: a clinicopathologic perspective for biomarker research. Alzheimers Dement.

[22] Haroutunian V, Purohit DP, Perl DP, Marin D, Khan K, Lantz M (1999). Neurofibrillary tangles in nondemented elderly subjects and mild Alzheimer disease. Arch Neurol.

[23] Iseki E, Tsunoda S, Suzuki K, Takayama N, Akatsu H, Yamamoto T (2002). Regional quantitative analysis of NFT in brains of non-demented elderly persons: comparisons with findings in brains of late-onset Alzheimer’s disease and limbic NFT dementia. Neuropathology.

[24] Taylor CR, Levenson RM (2006). Quantification of immunohistochemistry–issues concerning methods, utility and semiquantitative assessment II. Histopathology.

[25] Falk T, Mai D, Bensch R, Çiçek Ö, Abdulkadir A, Marrakchi Y (2019). U-Net: deep learning for cell counting, detection, and morphometry. Nat Meth Nat.

[26] Campanella G, Hanna MG, Geneslaw L, Miraflor A, Werneck Krauss Silva V, Busam KJ (2019). Clinical-grade computational pathology using weakly supervised deep learning on whole slide images. Nat Med.

[27] Wang S, Yang DM, Rong R, Zhan X, Xiao G (2019). Pathology image analysis using segmentation deep learning algorithms. Am J Pathol.

[28] Tang Z, Chuang KV, DeCarli C, Jin L-W, Beckett L, Keiser MJ (2019). Interpretable classification of Alzheimer’s disease pathologies with a convolutional neural network pipeline. Nat Commun.

[29] Signaevsky M, Prastawa M, Farrell K, Tabish N, Baldwin E, Han N (2019). Artificial intelligence in neuropathology: deep learning-based assessment of tauopathy. Lab Investig J Tech Meth Pathol.

[30] McKenzie AT, Marx G, Koenigsberg D, Sawyer M, Iida MA, Walker JM (2022). Interpretable deep learning of myelin histopathology in age-related cognitive impairment. Acta Neuropathol Commun.

[31] Lai Z, Wang C, Hu Z, Dugger BN, Cheung S-C, Chuah C-N (2021). A semi-supervised learning for segmentation of gigapixel histopathology images from brain tissues. Annu Int Conf IEEE Eng Med Biol Soc.

[32] Koga S, Ghayal NB, Dickson DW (2021). Deep learning-based image classification in differentiating tufted astrocytes, astrocytic plaques, and neuritic plaques. J Neuropathol Exp Neurol.

[33] Signaevsky M, Marami B, Prastawa M, Tabish N, Iida MA, Zhang XF (2022). Antemortem detection of Parkinson’s disease pathology in peripheral biopsies using artificial intelligence. Acta Neuropathol Commun.

[34] Wong DR, Tang Z, Mew NC, Das S, Athey J, McAleese KE (2022). Deep learning from multiple experts improves identification of amyloid neuropathologies. Acta Neuropathol Commun.

[35] Badrinarayanan V, Kendall A, Cipolla R (2017). SegNet: a deep convolutional encoder-decoder architecture for image segmentation. IEEE Trans Patt Anal Mach Intell.

[36] Morris JC (1997). Clinical dementia rating: a reliable and valid diagnostic and staging measure for dementia of the alzheimer type. Int Psychogeriatr.

[37] Seabold S, Perktold J (2010) Statsmodels: econometric and statistical modeling with python. Austin, Texas; 2010 [cited 2022 Apr 14]. p. 92–6. Available from: https://conference.scipy.org/proceedings/scipy2010/seabold.html

[38] Wickham H (2016) ggplot2: elegant graphics for data analysis. 2nd ed. 2016. Cham: Springer International Publishing : Imprint: Springer

[39] Nelson PT, Jicha GA, Schmitt FA, Liu H, Davis DG, Mendiondo MS (2007). Clinicopathologic correlations in a large Alzheimer disease center autopsy cohort: neuritic plaques and neurofibrillary tangles “do count” when staging disease severity. J Neuropathol Exp Neurol.

[40] Koga S, Parks A, Kasanuki K, Sanchez-Contreras M, Baker MC, Josephs KA (2017). Cognitive impairment in progressive supranuclear palsy is associated with tau burden. Mov Disord Off J Mov Disord Soc.

[41] Giannakopoulos P, Herrmann FR, Bussière T, Bouras C, Kövari E, Perl DP (2003). Tangle and neuron numbers, but not amyloid load, predict cognitive status in Alzheimer’s disease. Neurology.

[42] Alosco ML, Cherry JD, Huber BR, Tripodis Y, Baucom Z, Kowall NW (2020). Characterizing tau deposition in chronic traumatic encephalopathy (CTE): utility of the McKee CTE staging scheme. Acta Neuropathol (Berl).

[43] Arezoumandan S, Xie SX, Cousins KAQ, Mechanic-Hamilton DJ, Peterson CS, Huang CY, et al. (2022) Regional distribution and maturation of tau pathology among phenotypic variants of Alzheimer’s disease. Acta Neuropathol (Berl) [Internet]. 2022 [cited 2022 Aug 23]; Available from: 10.1007/s00401-022-02472-x 10.1007/s00401-022-02472-x PMC9936795 35871112

[44] Kaufman SK, Svirsky S, Cherry JD, McKee AC, Diamond MI (2021). Tau seeding in chronic traumatic encephalopathy parallels disease severity. Acta Neuropathol (Berl).

[45] Cherry JD, Mez J, Crary JF, Tripodis Y, Alvarez VE, Mahar I (2018). Variation in TMEM106B in chronic traumatic encephalopathy. Acta Neuropathol Commun.

[46] Armstrong RA, McKee AC, Alvarez VE, Cairns NJ (2017). Clustering of tau-immunoreactive pathology in chronic traumatic encephalopathy. J Neural Transm.

[47] Edwards G, Zhao J, Dash PK, Soto C, Moreno-Gonzalez I (2020). Traumatic brain injury induces tau aggregation and spreading. J Neurotrauma.

[48] Cho H, Choi JY, Hwang MS, Kim YJ, Lee HM, Lee HS (2016). In vivo cortical spreading pattern of tau and amyloid in the Alzheimer disease spectrum: Tau and Amyloid in AD. Ann Neurol.

[49] Clavaguera F, Hench J, Goedert M, Tolnay M (2015). Invited review: Prion-like transmission and spreading of tau pathology: prion-like transmission and spreading of tau pathology. Neuropathol Appl Neurobiol.

[50] Fuster-Matanzo A, Hernández F, Ávila J (2018). Tau spreading mechanisms; implications for dysfunctional tauopathies. Int J Mol Sci..

[51] Maphis N, Xu G, Kokiko-Cochran ON, Jiang S, Cardona A, Ransohoff RM (2015). Reactive microglia drive tau pathology and contribute to the spreading of pathological tau in the brain. Brain.

[52] Medina M, Avila J (2014) The role of extracellular Tau in the spreading of neurofibrillary pathology. Front Cell Neurosci [Internet]. 2014 [cited 2022 Jun 5];8. 10.3389/fncel.2014.00113 10.3389/fncel.2014.00113 PMC4005959 24795568

[53] Demaegd K, Schymkowitz J, Rousseau F (2018). Transcellular spreading of Tau in tauopathies. ChemBioChem.

[54] Brunello CA, Merezhko M, Uronen R-L, Huttunen HJ (2020). Mechanisms of secretion and spreading of pathological tau protein. Cell Mol Life Sci.

[55] Sharma H, Zerbe N, Lohmann S, Kayser K, Hellwich O, Hufnagl P (2022) A review of graph-based methods for image analysis in digital histopathology. Diagn Pathol [Internet]. 2015 [cited 2022 Aug 23]; Available from: http://www.diagnosticpathology.eu/content/index.php/dpath/article/view/61

[56] Teylan M, Mock C, Gauthreaux K, Chen Y-C, Chan KCG, Hassenstab J (2020). Cognitive trajectory in mild cognitive impairment due to primary age-related tauopathy. Brain.

[57] Farrell K, Iida MA, Cherry JD, Casella A, Stein TD, Bieniek KF (2022). Differential vulnerability of hippocampal subfields in primary age-related tauopathy and chronic traumatic encephalopathy. J Neuropathol Exp Neurol..

[58] Walker JM, Richardson TE, Farrell K, Iida MA, Foong C, Shang P (2021). Early selective vulnerability of the CA2 hippocampal subfield in primary age-related tauopathy. J Neuropathol Exp Neurol.

[59] Wilson RS, Yu L, Trojanowski JQ, Chen E-Y, Boyle PA, Bennett DA (2013). TDP-43 pathology, cognitive decline, and dementia in old age. JAMA Neurol.

[60] Kapasi A, Yu L, Boyle PA, Barnes LL, Bennett DA, Schneider JA (2020). Limbic-predominant age-related TDP-43 encephalopathy, ADNC pathology, and cognitive decline in aging. Neurology.

